# Energy expenditure, oxygen consumption, and heart rate while exercising on seven different indoor cardio machines at maximum and self-selected submaximal intensity

**DOI:** 10.3389/fspor.2024.1313886

**Published:** 2024-02-08

**Authors:** Pablo Prieto-González, Fatma Hilal Yagin

**Affiliations:** ^1^ Sport Sciences and Diagnostics Research Group, GSD-HPE Department, Prince Sultan University, Riyadh, Saudi Arabia; ^2^ Department of Biostatistics and Medical Informatics, Faculty of Medicine, Inonu University, Malatya, Turkey

**Keywords:** Rate of Perceived Exertion, energy expenditure, heart rate, oxygen consumption, indoor cardio machines

## Abstract

**Objective:**

One of the main objectives of practicing indoor cardiovascular exercise is to maximize caloric expenditure. This study aimed to compare energy expenditure (EE), oxygen consumption (VO2), and heart rate (HR) recorded in middle-aged adults while exercising on seven different indoor cardiovascular machines at self-selected maximal and submaximal intensity.

**Method:**

Thirty recreational-active adult males (Age: 41.69 ± 4.64) performed 12-min bouts at RPE (Rate of perceived exertion) 17 and maximum intensity (MAX INT) on the following indoor cardio machines: Recumbent bike (r_BIKE), upright bike (u-BIKE), spin bike (s-BIKE), rowing machine (ROW), elliptical trainer (ELLIP), stair climber (STAIR), and treadmill (TMILL). Heart rate (HR) and oxygen consumption (VO2) were measured during exercise, whereas EE (energy expenditure) was calculated indirectly.

**Results:**

Overall, TMILL induced the highest levels of EE, VO2, and HR, followed by STAIR, ELLIP, s_BIKE, u_BIKE, ROW, and r_BIKE. RPE was reliable across exercise modalities (r_BIKE, u-BIKE, s-BIKE, ROW, ELLIP, STAIR, and TMILL) and intensities (RPE 17 and MAX INT) for EE, HR, and VO2 measurements.

**Conclusion:**

To maximize EE while performing indoor cardiovascular exercise for recreational active middle-aged male participants, the TMILL is the best option, followed by the STAIR and the ELLIP. The least recommended options are, respectively, s_BIKE, u_BIKE, ROW, and r_BIKE. Beyond caloric expenditure considerations, promoting exercises that participants genuinely enjoy can enhance adherence, fostering sustained health benefits. Furthermore, RPE is a reliable tool for assessing EE, VO2, and HR across different exercise modalities and intensities.

## 1 Introduction

Nowadays, a sedentary lifestyle and unhealthy diets are the two predominant triggers for infirmity, particularly non-communicable diseases (1). To minimize the risk these diseases and promote health and quality of life improvements, the practice of aerobic exercise is an essential factor. Aerobic exercise is defined as “a physical activity that uses large muscle groups, is rhythmic in nature, and can be sustained for at least 10 min”. The practice of aerobic physical exercise is related to numerous beneficial effects, both physical and psychological, among which it is worth highlighting: Improvement of cardiovascular health, reduction of the risk of suffering from diabetes, hypertension, coronary diseases, various types of cancer, maintenance of a healthy weight, reduction of anxiety and stress levels and improved self-esteem (2–4). By virtue of these benefits, the W.H.O. (World Health Organization) recommends that adults practice 150–300 min of aerobic physical activity of moderate intensity or 75–150 min of aerobic physical activity of vigorous intensity; or an equivalent combination of moderate and vigorous intensity activity weekly (2).

When it comes to practicing aerobic exercise, there are many options, such as running, swimming, cycling, rowing, skating, or skiing (5). However, the practice of outdoor physical activity is conditioned by the climatic conditions of each location. In this sense, there is a wide range of indoor fitness machines that allow individuals to practice of aerobic exercise throughout the year, such as treadmills, stationary bikes, ellipticals, stair climbers, or rowing machines. Therefore, sports science professionals must know the risks and benefits of each machine to advise each individual which modality they should select. Therefore, aspects such as the injuries each machine can cause, the coordination requirements, and the characteristics and preferences of each individual must be considered (6, 7). Nevertheless, apart from these aspects, a key factor in selecting one specific indoor machine is the energy consumption per unit of time for a given intensity. Not surprisingly, individuals who refrain from physical activity often attribute their inactivity to time constraints (8). In this context, optimizing exercise time, and consequently increasing energy expenditure, can prove beneficial for many individuals striving to attain fitness and health goals. This is particularly relevant for those aiming to maintain or reduce body weight and enhance body composition (9), recognizing that weight loss programs involving aerobic exercise typically necessitate long-term intervention (10).

In this context, various studies have been devoted to compare the energy expenditure (EE) of different indoor machines in the last four decades. However, some included a reduced number of exercise modalities (6, 11–14) since some indoor machines have become popular after the publication of those research (15). Similarly, within the published works, there are conflicting results between studies. Significant differences were observed in energy consumption between the different modalities (12, 13, 16–20), whereas in other cases, there are not (6, 11, 14). Moreover, there are also significant discrepancies regarding the experimental protocols used in the studies. In some research, participants performed exercise bouts of 4–5 min (13, 17, 20), whereas in other studies, the bouts lasted 15 min (6, 18). In this regard, it is necessary to mention that when the duration is between 4 and 5 min, the contribution of anaerobic metabolism is significant. Thus, using exercise bouts longer than 10 min would be more suitable to assess aerobic efficiency due to the lower contribution of anaerobic metabolism (21–23).

Importantly, in those studies where energy consumption in different indoor exercise machines was measured, the protocols involved using self-selected exercise intensities corresponding to submaximal RPE (Rating of Perceived Exertion) values, commonly between 11 and 15 on the Borg 6–20 scale. However, the problem is that using RPE as a parameter to estimate exercise intensity is controversial. Several authors consider that subjects have difficulties integrating psycho-physical elements (18, 24). Therefore, they suggest using RPE cautiously and argue that the relationship between physiological variables and RPE should be further investigated (18, 24). Thus, while in some studies it was found that RPE is a valid indicator of exercise intensity (13, 25), Dumbar et al. verified that RPE validity levels are reduced when exercising on treadmills at high intensities, but not on cycle ergometers (26). For this reason, it has been proposed to combine RPE with other methods (27).

Finally, there are two other important aspects of the studies aimed at analyzing energy consumption on indoor exercise machines. Research has been conducted preferably with college-age individuals rather than middle-aged adults (6, 14, 17, 20), and in some published works, the study participants were either familiar with the machines or untrained individuals (6, 17). In this context, new research regarding the energy consumption in different indoor cardio machines is warranted, and potential strengths and limitations of previous studies must be considered. Therefore, the present study aimed to compare the EE, oxygen consumption (VO2), and heart rate (HR) registered in middle age adults while exercising on seven different indoor cardio machines at maximum and self-selected submaximal intensity.

## 2 Materials and methods

A prospective study using a repeated measures design was conducted.

### 2.1 Subjects

A total of 30 recreational-active adult males aged between 35 and 49 were included in the present study (see descriptive data in Table 1). All of them had more than 10 years of experience in the practice of cardio exercise with indoor machines. They had practiced on regular basis a minimum of two cardiovascular exercise sessions with indoor machines per week (of 30–60 min per session), during the last 10 years. They were accustomed to exercising regularly on the following seven indoor cardio machines: Recumbent bike (r_BIKE), upright bike (u-BIKE), spin bike (s-BIKE), rowing machine (ROW), elliptical trainer (ELLIP), stair climber (STAIR), and treadmill (TMILL). None of them suffered from cardiovascular, respiratory, neuromuscular, or metabolic diseases or pathologies incompatible with the practice of physical exercise. They were informed that they could leave the study without penalty. None of the study participants received compensation for participating in the study. They were duly informed of the purpose, risks, and benefits of participating in the research. They signed an informed consent document expressing their willingness to be included. The study was conducted in accordance with the ethical principles outlined in the declaration of Helsinki and was approved by the institutional review board of Prince Sultan University, Saudi Arabia (PSU IRB-2023-11-0163).

**Table 1:** Characteristics of the study participants.

Age (years)	41.69 ± 4.64
Sex	Male
Height (m)	1.78 ± 0.06
Body mass (Kg)	73.13 ± 7.87
Body mass index (kg/m^2^)	23.04 ± 1.46
VO2max (ml/kg/min)*	57.34 ± 3.12
TMHR (bpm)^#^	178.81 ± 3.31

* VO2max was calculated with the equation: VO2max = (22.351 × kilometers) − 11.288 (57), where “kilimeters” represent the distance covered by the athletes in the 12-min bout at maximum intensity on the treadmill.

^#^ TMHR (Theoretical maximum heart rate) of the subjects was calculated with the equation: HRmax = 208 − (0.7 × age) (58–60). Data were presented as mean ± standard deviation.

### 2.2 Experimental design

The seven indoor cardio machines used in the present study were: One r_BIKE (SportsArt G545R, Mukilteo, Washington, USA), one u-BIKE (SportsArt G545U, Mukilteo, Washington, USA), one s_BIKE (Livestrong® LS9.9IC, Austin, Texas, United States), one ROW (Concept2 RowErg®, Minneapolis, Minnesota, United States), one ELLIP (SportsArt E840, Mukilteo, Washington, USA), one STAIR (Life Fitness Integrity CLSS, Rosemont, Illinois, USA), and one TMILL (Cybex 625T, Rosemont, Illinois, USA). Prior to the commencement of the intervention, a habituation period of four weeks was carried out. Three weekly sessions were held on alternate days. The study participants received precise information regarding the proper form while exercising on the mentioned seven indoor machines. Particular emphasis was placed on stride frequency and length on the TMILL, rpm (revolutions per min) on the three different bikes and ELLIP, and the number of strokes per min on the ROW. For this purpose, the current scientific evidence in this regard was considered (28–34). Study participants also received information regarding the use of the RPE based on the current guidelines (35).

Once the habituation period was completed, the intervention was carried out for five weeks. It consisted of 14 sessions (three per week), with a rest period of 48 h between sessions. All sessions were carried out at the same time (between 12.00 pm and 2.00 pm) at a constant temperature of 22 °C. The study participants were instructed not to eat 3 h before performing the exercise activities. They were asked not to ingest caffeine or supplements. They were also requested not to alter their diet and physical activity habits. The subjects exercised in each session 12 min on one of the seven indoor machines (r_BIKE, v-BIKE, s-BIKE, ROW, ELLIP, STAIR, and TMILL) at one of two intensities used in the study: RPE 17 (which corresponds to “very hard” in the 6–20 Borg Scale), and maximum intensity (MAX INT) (36). MAX INT was included alongside with RPE since the reliability of RPE in submaximal assessments has been previously questioned in some studies (19, 25). This dual approach aimed to improve control and consistency in measuring participants' effort. The assignment to each machine and intensity level was made randomly and equitably (i.e., Day 1: Two study participants exercised on the recumbent bike at RPE 17, two study participants exercised on the recumbent bike at MAX INT, two study participants exercised on the upright bike at RPE 17, two study participants exercised on the upright bike at MAX INT…). In each session, study participants underwent a well-structured 10-min warm-up properly divided into two phases (37). This warm-up commenced with a 5-min cardio exercise tailored to the individual, utilizing the designated exercise machine. Following this initial cardio phase, participants devoted 5 min to dynamic joint mobilization, following a cephalocaudal pattern. Subsequently, each participant performed a12-min cardio bout using their designated exercise machine, adhering to the prescribed intensity level. There were explicitly instructed to sustain a steady pace, and also received verbal encouragement to ensure they maintained the requisite intensity (38, 39). The measurement of the metabolic and cardiorespiratory parameters was carried out in the last 5 minu of the bout. HR was assessed with a HR monitor Polar H10 pulsometer (Kempele, Finland), VO2 with a Cosmed K4b2 gas exchange analyzer (Rome, Italy), and weight, height and BMI (body mass index) with a Seca digital column scale, model 769 (Hamburg, Germany). Anthropometric measurements were conducted with the study participants barefoot. Body mass was measured to the nearest 0.1 kg, and height to the nearest 0.1 cm. Finally, taking into account that the exercise underwent was performed at steady pace, the energy expenditure was calculated in Kcal using the Weir formula, considering the nonprotein respiratory quotient, and on the basis that the caloric equivalent of oxygen is 4.84 kcal per every liter of oxygen consumed (40, 41).

### 2.3 Statistical analysis

Data are presented as mean ± standard deviation. The assumptions of normality and sphericity were verified using the Shapiro–Wilk and Mauchly tests, respectively. When the sphericity condition was not met, the Greenhouse-Geisser, Huynh-Feldt, and lower bound adjustments were applied. To assess the consistency of the measurements, the intraclass correlation coefficient (ICC) between exercise modalities and intensities was calculated. Values below 0.5 were interpreted as poor reliability, between 0.5 and 0.75, moderate reliability, between 0.75 and 0.9, good reliability, and between 0.90 and 1, excellent reliability (42). To examine the possible existence of differences between exercise modalities (r_BIKE vs. v-BIKE vs. s-BIKE vs. ROW vs. ELLIP vs. STAIR vs. TMILL) at a given intensity (RPE 17 vs. MAX INT) in terms of HR frequency, VO2, and EE, the one-way repeated measures ANOVA test was performed, with Bonferrony's test for *post hoc* comparisons. The effect size was calculated using the partial eta squared parameter (*η*^2^*_p_*). Values of *η*^2^*_p_* = 0.01 were interpreted as small effect, *η*^2^*_p_* = 0.06 medium effect, and *η*^2^*_p_* = 0.14 large effect (43). The significant level was set at *p* ≤ 0.05. The statistical analysis was carried out with the IBM SPSS statistics program, version 26 (Chicago, USA).

## 3 Results

The ICC obtained between exercise modalities for EE, HR, and VO2 was, in all cases, greater than 0.9 (see Table 2). This reflects excellent reliability. Similarly, the ICC for energy expenditure between exercise intensities for HR, VO2, and EE was also greater than 0.9 in all cases, thus reflecting excellent reliability (see Tables 3–5).

**Table 2:** Intraclass correlation coefficient between exercise modalities (r_BIKE, v-BIKE, s-BIKE, ROW, ELLIP, STAIR, and TMILL) at different intensities (RPE 17 and MAX INT).

Variable	ICC	95% interval confidence	Sig
EE (RPE 17)	0.978	0.957–0.988	*p* < 0.001
HR (RPE 17)	0.923	0.855–0.961	*p* < 0.001
VO2 (RPE 17)	0.954	0.913–0.976	*p* < 0.001
EE (MAX INT)	0.963	0.929–0.981	*p* < 0.001
HR (MAX INT)	0.951	0.907–0.975	*p* < 0.001
VO2 (MAX INT)	0.929	0.866–0.964	*p* < 0.001

EE, energy expenditure; HR, heart rate; VO2, oxygen consumption; RPE, rating of Perceived Exertion; MAX INT, maximum intensity; ICC, intraclass correlation coefficient; Sig, level of significance.

**Table 3:** Intraclass correlation coefficient for heart rate between exercise intensities (RPE 17 and MAX INT) within the same exercise mode.

Machine	ICC	95% interval confidence	Sig
r_BIKE	0.934	0.875–0.966	*p* < 0.001
u_BIKE	0.974	0.950–0.986	*p* < 0.001
s_BIKE	0.969	0.941–0.984	*p* < 0.001
ROW	0.981	0.963–0.990	*p *< 0.001
ELLIP	0.972	0.946–0.985	*p* < 0.001
STAIR	0.916	0.842–0.957	*p* < 0.001
TMILL	0.957	0.918–0.978	*p *< 0.001

r_BIKE, recumbent bike; u-BIKE, upright bike; s-BIKE, spin bike; ROW, rowing machine; ELLIP, elliptical trainer; STAIR, stair climber; TMILL, treadmill; ICC, intraclass correlation coefficient; Sig, level of significance.

**Table 4:** Intraclass correlation coefficient for oxygen consumption between exercise intensities (RPE 17 and MAX INT) within the same exercise mode.

Machine	ICC	95% interval confidence	Sig
r_BIKE	0.932	0.872–0.965	*p* < 0.001
u_BIKE	0.956	0.916–0.977	*p* < 0.001
s_BIKE	0.954	0.913–0.976	*p* < 0.001
ROW	0.967	0.937–0.983	*p* < 0.001
ELLIP	0.957	0.918–0.978	*p* < 0.001
STAIR	0.906	0.823–0.952	*p* < 0.001
TMILL	0.942	0.890–0.970	*p* < 0.001

r_BIKE, recumbent bike; u-BIKE, upright bike; s-BIKE, spin bike; ROW, rowing machine; ELLIP, elliptical trainer; STAIR, stair climber; TMILL, treadmill; ICC, intraclass correlation coefficient; Sig, level of significance.

**Table 5:** Intraclass correlation coefficient for energy expenditure between exercise intensities (RPE 17 and MAX INT) within the same exercise mode.

Machine	ICC	95% interval confidence	Sig
r_BIKE	0.927	0.862–0.963	*p* < 0.001
u_BIKE	0.963	0.929–981	*p* < 0.001
s_BIKE	0.952	0.909–975	*p* < 0.001
ROW	0.965	0.933–982	*p* < 0.001
ELLIP	0.956	0.916–977	*p* < 0.001
STAIR	0.911	0.832–954	*p* < 0.001
TMILL	0.941	0.888–970	*p* < 0.001

r_BIKE, recumbent bike; u-BIKE, upright bike; s-BIKE, spin bike; ROW rowing machine, ELLIP, elliptical trainer; STAIR, stair climber; TMILL, treadmill; ICC, intraclass correlation coefficient; Sig, level of significance.

**Figure 1:**
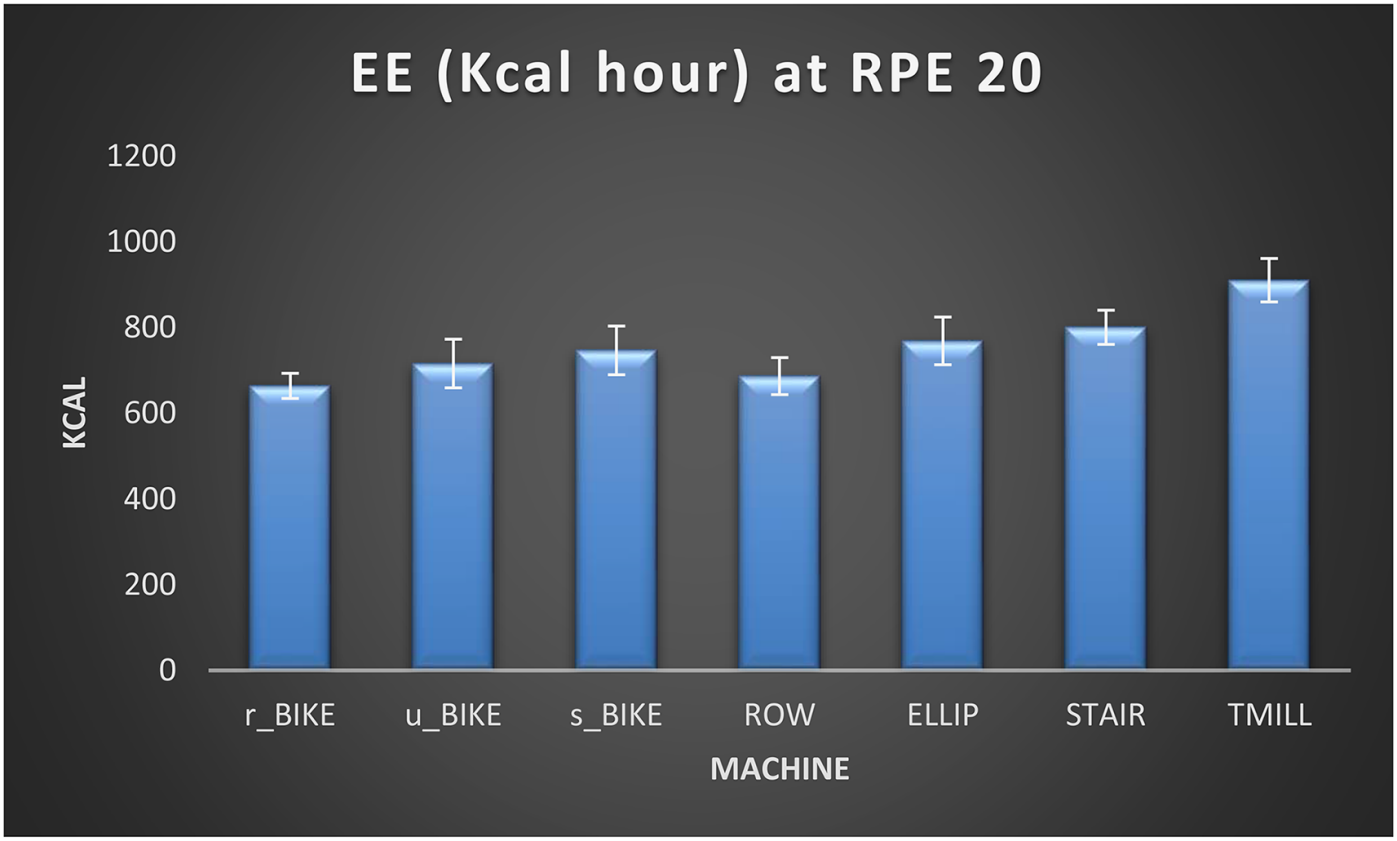
Energy expenditure (Kcal/h) in each of the seven exercise modalities at RPE 17. EE (energy expenditure), r_BIKE (Recumbent bike), u-BIKE (upright bike), s-BIKE (spin bike), ROW (rowing machine), ELLIP (elliptical trainer), STAIR (stair climber), TMILL (treadmill).

**Figure 2:**
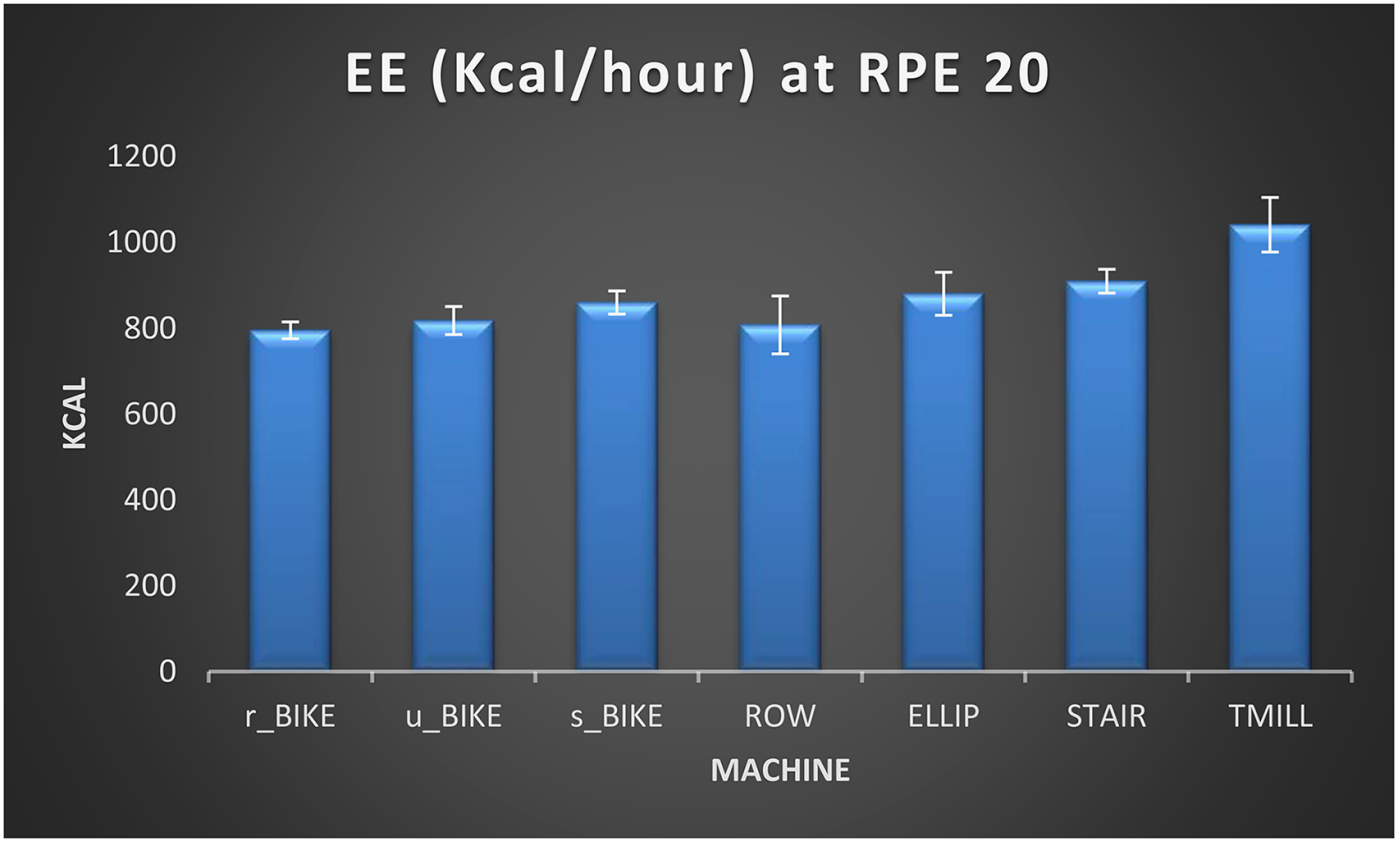
Energy expenditure (Kcal/h) in each of the seven exercise modalities at RPE 20. EE (energy expenditure), r_BIKE (Recumbent bike), u-BIKE (upright bike), s-BIKE (spin bike), ROW (rowing machine), ELLIP (elliptical trainer), STAIR (stair climber), TMILL (treadmill).

As for the comparisons between exercise modalities, the one-way ANOVA test determined the existence of significant differences between exercise modalities for EE at RPE17 [*F*(6,174) = 855.70; *p* < 0.001; *η*^2^*_p_* = .951]. After that, the *post hoc* analysis revealed the existence of significant differences between all exercise modalities (*p* ≤ 0.05), being r_BIKE the machine producing the lowest EE, followed by ROW, v-BIKE, s-BIKE, ELLIP, STAIR, and TMILL (see Table 6). At MAX INT, the one-way ANOVA test indicated the existence of significant differences between exercise modalities for EE [*F*(6,174) = 678.36; *p* < 0.001; *η*^2^*_p_* = .927]. Then, the subsequent *post hoc* analysis showed that the EE of the TMILL was significantly higher than that of the STAIR (*p* < 0.001), the EE of the STAIR higher than that of the ELLIP (*p* < 0.001), the EE of the ELLIP higher than that of the s_BIKE (*p* < 0.001), the EE of the s_BIKE higher than that of the ROW (*p* < 0.001), and the EE of the u_BIKE higher than that of the r_BIKE (*p* < 0.001). However, no significant differences in EE were detected between ROW and r_BIKE and ROW and u_BIKE (see Table 6). Likewise, Figures 1, 2 show the EE per hour that can be attained by exercising at RPE 17 and INT MAX respectively in each of the seven exercise modalities included in this study.

**Table 6:** Energy expenditure (kcal/min), heart rate (bpm), and oxygen consumption attained by the study participants at RPE17 and maximum intensity.

Machine	Energy expenditure (kcal/min)		Heart rate (bpm)		Oxygen consumption (ml/kg/min)	
	RPE17	MAX INT	RPE17	MAX INT	RPE17	MAX INT
r_BIKE	11.07 ± 0.41^1^	13.25 ± 0.32^a^	129.26 ± 3.05^1^	145.43 ± 2.41^a^	31.23 ± 1.59^1^	37.36 ± 0.9^a^
u-BIKE	11.94 ± 0.50^2^	13.63 ± 0.54^b^	135.73 ± 3.72^2^	148.31 ± 4.03^b^	33.68 ± 1.41^2^	38.45 ± 1.51^b^
s-BIKE	12.45 ± 0.40^3^	14.33 ± 0.44^c^	139.53 ± 3.01^3^	153.46 ± 3.32^c^	35.13 ± 1.13^3^	40.41 ± 1.24^c^
ROW	11.45 ± 0.94^4^	13.46 ± 1.12^d^	132.03 ± 7.03^4^	147.03 ± 8.35^d^	32.28 ± 2.67^4^	37.97 ± 3.11^d^
ELLIP	12.82 ± 0.95^5^	14.67 ± 0.83^e^	142.23 ± 7.08^5^	155.96 ± 6.21^e^	36.15 ± 2.68^5^	41.36 ± 2.31^e^
STAIR	13.35 ± 0.66^6^	15.16 ± 0.46^f^	146.16 ± 4.94^6^	159.63 ± 3.42^f^	37.64 ± 1.87^6^	42.76 ± 1.28^f^
TMILL	15.18 ± 0.84	17.35 ± 1.05	159.76 ± 6.27	175.91 ± 7.86	42.81 ± 2.38	48.93 ± 2.94

r_BIKE, recumbent bike; u-BIKE, upright bike; s-BIKE, spin bike; ROW, rowing machine; ELLIP, elliptical trainer; STAIR, stair climber; TMILL, treadmill; RPE, rating of Perceived Exertion; MAX INT, maximum intensity.

^1^ Significantly lower than ROW.

^2^ Significantly lower than s_BIKE.

^3^ Significantly lower than ELLIP.

^4^ Significantly lower than u_BIKE.

^5^ Significantly lower than STAIR.

^6^ Significantly lower than TMILL.

^a^ Significantly lower than ROW.

^b^ Significantly lower than s_BIKE.

^c^ Significantly lower than ELLIP.

^d^ Significantly lower than u_BIKE.

^e^ Significantly lower than STAIR.

^f^ Significantly lower than TMILL.

*p* ≤ 0.05.

Moreover, the one-way ANOVA test determined the existence of significant differences between exercise modalities for HR at RPE17 [*F*(6,174) = 831.70; *p* < 0.001; *η*^2^*_p_* = .966] and MAX INT [*F*(6,174) = 722.65; *p* < 0.001; *η*^2^*_p_* = .946]. Then, the *post hoc* analysis revealed the existence of significant differences between all exercise modalities (*p* ≤ 0.05), being r_BIKE the machine producing the lowest EE, followed by ROW, u_BIKE, s_BIKE, ELLIP, STAIR, and TMILL (see Table 6).

Finally, the one-way ANOVA test showed the existence of significant differences between exercise modalities for VO2 at RPE17 [*F*(6,174) = 645.19; *p* < 0.001; *η*^2^*_p_* = .931]. Then, the subsequent *post hoc* analysis revealed that the VO2 of the TMILL was significantly higher than that of the STAIR (*p* < 0.001), the VO2 of the STAIR higher than that of the ELLIP (*p* < 0.001), the VO2 of the ELLIP and s_BIKE (*p* < 0.001) higher than that of the u_BIKE, the VO2 of the u_BIKE higher than that of the ROW and r_BIKE (*p* < 0.001). Nevertheless, no significant differences in VO2 were detected between ELLIP and u_BIKE, and between ROW and r_BIKE (see Table 6). At MAX INT, the one-way ANOVA test also determined the existence of significant differences between exercise modalities for VO2 [*F*(6,174) = 513.36; *p* < 0.001; *η*^2^*_p_* = .919]. Then, the subsequent *post hoc* analysis revealed that the VO2 of the TMILL was significantly higher than that of the STAIR (*p* < 0.001), the EE of the STAIR higher than that of the ELLIP (*p* < 0.001), the EE of the ELLIP higher than that of the s_BIKE (p < 0.001), the EE of the s_BIKE higher than that of the ROW (*p* < 0.001), and the EE of the u_BIKE higher than that of the r_BIKE (*p *< 0.001). In contrast, no significant differences in EE were detected between ROW and r_BIKE, and ROW and u_BIKE (see Table 6).

## 4 Discussion

This research aimed to compare the EE, VO2, and HR registered in middle-aged adults while exercising on seven indoor cardio machines at RPE 17 and MAX INT. The results showed that the TMILL induced higher HR levels, followed by the STAIR, ELLIP, s_BIKE, u_BIKE, ROW, and r_BIKE. Similar results were recorded for EE and V02, with the exception that at MAX INT, there were no significant differences in EE and VO2 between ROW, r_BIKE, and u_BIKE, and at RPE 17, no significant differences were observed between s_BIKE and ELLIP. These results agree with those that Huang (2018) and Jensen et al. (2019) obtained. In both cases, it was also verified that TMILL induced higher HR and EE, respectively, followed by STAIR, ELLIP, u_BIKE, ROW, and r_BIKE (20, 44), despite the machineś brand and characteristics differed in some cases. The results of the present study also agree to a large extent with those registered by Zeni et al. (1996a) and Zeni et al. (1996b) (45, 46). The main discrepancies are that the HR and EE values obtained on ROW were higher than those of the cycle ergometer, unlike in our study.

Based on the results obtained in the present study, we consider that the higher HR, VO2, and EE induced by the TMILL compared to the other indoor machines is because it is a high-impact exercise that activates numerous muscle groups, including large muscles such as rectus and biceps femoris, which implies a higher metabolic and cardiovascular demand (13, 47, 48). Similarly, another potential explanation for the elevated HR, VO2, and EE on the TMILL may be that, unlike the STAIR and the ELLIP, the performer's movement is not restricted by a predetermined route that externally influences stride frequency and length. And this factor can be even more relevant when exercising at high intensities (20).

The STAIR was the second exercise modality that induced higher values in terms of HR, VO2, and EE. In this machine, a movement simulating climbing stairs is performed. Subjects push down the steps exerting force with a strength that depends on their body weight, strength, and coordination. By increasing the difficulty level, the pedals retain subjectś body weight to a lesser extent, so they are forced to increase step frequency (48). This aspect, together with the fact that applying force in the vertical vector is harder than in the horizontal vector, could be the reasons why the STAIR elicited higher HR, VO2, and EE than the ELLIP (48), despite the STAIR used in this research does not have movable handles for the arms, (which implies less energy expenditure) (48). Another possible reason why ELLIP yielded lower HR, VO2, and EE values than TMILL and STAIR is that on the ELLIP, the hip flexor and extensor muscles (i.e., gluteus maximus, tensor fascia latae) are preferentially utilized (49), whereas on the TMILL and STAIR, the knee flexors and extensors (i.e., quadriceps and hamstrings), which are larger, which are larger and effectively stimulated by the movement parameters of the exercise devices (47, 50).

Moreover, it is conceivable that the elliptical induced higher HR, VO2, and EE values than the three bicycles and the ROW used in the present study due to the simultaneous use of arms and legs on the ELLIP (unlike the bikes) and because the standing position requires greater physical exertion (18, 47–49). In turn, we consider that s_BIKE produced higher HR, VO2, and EE levels than u_BIKE and r_BIKE because spin bikes are designed to simulate the position and real cycling conditions of roads bike and have various adjustments to achieve an optimal posture to apply force (48). In addition, unlike stationary bikes (equipped with freewheels that allow the subjects to stop pedaling), spinning bikes have a flywheel, direct drive, and magnetic resistance (48) that favor more accurate resistance selection and pedaling. Therefore, subjects can use higher cadence and overcome greater resistance, which increases muscle activation (51). The u_BIKE also elicited higher HR, VO2, and EE values than the r_BIKE. The reason could be the position adopted by the subject on each machine. While the u_BIKE has no back rest, on the r_BIKE the subject adopts a reclined seated position (48), which requires applying less effort because, with less muscle activation, a more effective push against the selected resistance is exerted (20).

Finally, as for the ROW, we consider that it induced lower HR, VO2, and EE values than the TMILL, STAIR, ELLIP due to the sitting position. Since ROW is not a weight-bearing machine, cardiac and energy expenditure would be lower (52). The HR, VO2, and EE values obtained in the ROW were also lower than on the s_BIKE, and with some exceptions, than on the u_BIKE, despite the non-participation of the arms on the bikes. The reason could be that cycling is an activity of greater reciprocation than the ROW. That is to say, there is an almost permanent intervention of the muscles of both lower extremities (53), which could imply a higher EE and HR. In contrast, on the ROW, the duration of the recovery action is longer. Thus, a lower application of force is required during this phase, which could result in lower EE and HR (54). As for the comparison between ROW and r_BIKE, the lower values of EE, VO2, and HR observed in the latter could be due to the laid-back position, which, as previously mentioned, may facilitate a more efficient push, and therefore, the performance of a lesser effort. Nevertheless, based on the results of this study and previous research, it can be assumed that cycle ergometers and ROW induce similar EE, HR, and VO2 values. In this regard, Hagerman et al. (1988) conducted one research where participants produced more watts on the cycle ergometer than on the ROW, whereas the ROW elicited higher HR and Vo2max values (12).

Aside from the comparisons across exercise modalities, the results of the present study only partially agree with the studies by Kim et al. (2008) and Moyna et al. (2001) (16, 18). In the first case, the authors found that ELLIP induced a greater EE than TMILL and Airdine, whereas the cycle ergometer was the machine that produced the lowest EE. Based on these results, they consider that the discrepancies between the outcomes obtained in their study and others could be because perceptually-based exercise could not be reliable across indoor exercise machines (18). Another factor that could have conditioned their results is that the study participants were obese. As for the results obtained by Moyna et al. (2001), the EE induced by the TMILL and ski simulator in men was superior to the other machines (cycle ergometer, rowing ergometer, and rider). However, they also observed that the similar HR between the machines was higher in men than in women (16). In this case, although the reasons for the absence of significant differences between exercise modalities are not entirely clear, some authors consider that specific factors can alter the results in those studies aimed to compare the HR and EE between exercise modalities. The mentioned factors could include (13, 17, 55): (a) Level of familiarity with exercise machines, which was not controlled in some studies. (b) Study participants could be less familiar with certain machines, particularly those recently incorporated into the fitness field, such as ellipticals. (c) The amount of concentric and eccentric muscle activity performed on each machine. (d) Excitatory or inhibitory reflexes due to the development of reciprocal innervation pathways. (e) The characteristics of the same machine manufactured by different companies may vary. Thus, the EE, VO2, and HR elicited on treadmills may differ depending on its belt's stiffness.

The results of our study do not agree with those obtained in three previous research. Thus, Thomas et al. (1989) found no significant differences in VO2 between stationary cycle, ROW, ski simulator, and TMILL (walking) (14). However, in this study, only five subjects participated (with an average age of 23 years), and this circumstance could have conditioned the results. Similarly, Green et al. (2004) observed no significant differences in HR between TMILL and ELLIP modalities (17). The authors attribute this result to the fact that exercising on the ELLIP involves performing a less common movement pattern than running, and because the degree of familiarity of the subjects with the machines was not controlled. Additionally, Brown et al. (2010) did not register significant differences in EE between TMILL and ELLIP (6). The reason could be that the study participants were untrained college-aged subjects, and cadence was not controlled in the ELLIP.

Another noteworthy aspect of the present study is the high ICC observed between exercise modalities (r_BIKE, u-BIKE, s-BIKE, ROW, ELLIP, STAIR, and TMILL) and exercise intensities (RPE 17 and MAX INT) for EE, HR, and VO2 measurements. This finding reinforces the evidence obtained in previous studies where the RPE reliability to select exercise intensity on indoor cardio machines was demonstrated (13, 25). However, as for the RPE validity, certain research has also verified that RPE, in some cases, is not an accurate instrument to determine exercise intensity (26, 56). Dunbar et al. (1992) concluded that RPE is inaccurate when used in TMILL at high intensities (26), whereas Muyor (2013) observed that Borg and OMNI scales present low validity in estimating exercise intensity in indoor cycling (56). Therefore, future studies are warranted to analyze the RPE validity to select exercise intensity on indoor cardio modalities.

Finally, the strengths and limitations of the present study must be mentioned. Only seven indoor cardio modalities were included to ensure that all study participants were familiar with all the machines selected for the intervention protocol. This is a strength in terms of validity of the results, but also a weakness, as there are currently numerous indoor cardio machines available on the market. Another limitation was that the study participants were exclusively male. Thus, future studies might incorporate newer indoor cardio machines, including both males and females, but also ensure that the familiarization of the study participants with the machines does not condition the results. Moreover, instead of conducting maximal exercise tests on a treadmill to directly measure VO2max and HRmax, we employed an indirect approach. VO2max was calculated using a designated equation derived from the distance covered during the 12-min treadmill test. Similarly, rather than directly obtaining HRmax values, we utilized the theoretical maximum heart rate calculated through a designated formula for this purpose.

## 5 Conclusion

When exercising on indoor cardio machines at RPE 17 and maximum intensity, our findings reveal that the treadmill elicits the highest energy expenditure, oxygen consumption, and heart rate values. Following this physiological analysis, it is crucial to acknowledge the importance of enjoyment in exercise participation, as it is linked to adherence and overall health improvement, as described in the introduction. Therefore, for middle-aged males aiming to maximize caloric expenditure during indoor cardio exercise, the treadmill emerges as the primary choice. However, considering the significant role of consistency in exercise adherence and overall health maintenance, it is essential to encourage participants to engage with any of the tested exercises that they genuinely enjoy. This recommendation recognizes the individual preferences and enjoyment factor, which can contribute to sustained exercise participation, leading to long-term health benefits.

In the absence of specific conditioning factors such as injuries or strong personal preferences, the treadmill can still be considered the first option, followed by the stair stepper and the elliptical. Conversely, the upright bike, rowing machine, and recumbent bike are less recommended options for a single exercise session. Moreover, our study underscores the reliability of Rate of Perceived Exertion (RPE) as a valuable tool for assessing energy expenditure, oxygen consumption, and heart rate across various exercise modalities and intensities, particularly at very high and maximum intensity levels.

## 6 Practical applications

To optimize caloric expenditure in indoor cardiovascular exercise, the treadmill emerges as the most efficient modality, closely followed by the stair stepper and elliptical. Meanwhile, the spinning bike and upright bike are intermediate choices in terms of effectiveness for calorie burning. In contrast, the rowing machine (ROW) and recumbent bike (r_BIKE) prove to be less adept at achieving calorie-burning objectives during indoor cardio sessions.

Integral to these considerations is a nuanced understanding of the diverse movement patterns and muscle activation associated with each machine. The treadmill, engaging multiple muscle groups, imposes heightened metabolic and cardiovascular demands. Conversely, the elliptical focuses on engaging the hip flexor and extensor muscles.

Furthermore, the Rate of Perceived Exertion (RPE) serves as a reliable tool to assess exercise intensity across various modalities and intensities. This subjective measure of effort provides a practical framework for individuals to regulate their workouts, allowing for adjustments in exercise intensity based on their personal sensations throughout the session.

## Data Availability

The raw data supporting the conclusions of this article will be made available by the authors, without undue reservation.
